# The prevalence of coronary artery disease in rheumatoid arthritis patients in Palestine: a cross-sectional study

**DOI:** 10.1186/s41927-026-00613-3

**Published:** 2026-01-20

**Authors:** Rami Shrouf, Aleen Aldabbas, Razan Sobeih, Talal Asafrah, Sameh Issa, Dunia Salhab, Osama Ewidat, Nuha Riyad, Mohammed Alzer’e, Abrar Khdour, Ahmad Fasfoos, Saed Atawnah

**Affiliations:** 1 Faculty of Medicine, Hebron University, Hebron, Palestine; 2 Faculty of Medicine, An-Najah National University, Nablus, Palestine; 3 Faculty of Medicine, Al-Quds University, Abu-Dis, Palestine; 4 Rheumatologist, Ahli Hospital, Hebron, Palestine

**Keywords:** Rheumatoid arthritis, Coronary artery disease, Cardiovascular risk factors, Systemic inflammation, Palestine, Cross-sectional study

## Abstract

**Background:**

Rheumatoid arthritis (RA) is a systemic inflammatory disorder associated with a significantly increased risk of cardiovascular events. This study aims to assess the prevalence of coronary artery disease (CAD) and its associated risk factors among Palestinian patients with RA, a population for which this data has been lacking.

**Methods:**

A cross-sectional study was conducted from March to September 2024 at multiple rheumatology clinics in the West Bank, Palestine. The study included 384 patients with a confirmed RA diagnosis based on the American College of Rheumatology (ACR)/European League Against Rheumatism (EULAR) criteria. Data were collected on demographic characteristics, traditional cardiovascular risk factors, and RA-specific factors, including disease activity measured by the Disease Activity Score 28 (DAS28) and C-reactive protein (CRP) levels. Multivariable analysis was used to identify independent predictors of CAD.

**Results:**

The prevalence of CAD in this cohort was 25.5%. Multivariable analysis revealed that CAD was independently predicted by increasing age, dyslipidemia, and a first-degree family history of CAD. Markers of systemic inflammation, specifically higher disease activity (DAS28 Prevalence Ratio = 1.309) and elevated CRP levels (PR up to 2.108 for levels > 10 mg/L), also emerged as potent and independent predictors. Furthermore, a paradoxical, non-linear association was observed with anti-CCP antibody status, where low-positive titers conferred the highest risk (PR = 1.811), and a modest inverse association with BMI was noted (PR = 0.992), consistent with the ‘obesity paradox’.

**Conclusion:**

This study reveals a high prevalence of CAD among Palestinian patients with RA, driven by both traditional metabolic risk factors and RA-related systemic inflammation. The findings highlight an urgent need to integrate proactive cardiovascular disease prevention into the standard of care for RA in Palestine, recognizing RA as a cardiovascular risk-equivalent condition.

**Supplementary Information:**

The online version contains supplementary material available at 10.1186/s41927-026-00613-3.

## Background

Rheumatoid arthritis (RA) is a systemic autoimmune disorder marked by a chronic inflammatory process that primarily affects the joints [1],. Clinically, it often presents with a gradual onset of pain and swelling in small joints, typically symmetrically [2]. However, RA’s impact extends beyond the joints, with potential extra-articular manifestations affecting the heart, kidneys, lungs, and other organs [1]. The worldwide prevalence of RA is estimated to be approximately 0.46% [3].

A significant body of evidence highlights that RA is a major risk factor for cardiovascular diseases (CVDs), which are the primary cause of death globally [4]. Cardiovascular disease is a prevalent and serious complication of RA [5]. Meta-analyses have shown that patients with RA have a 48% higher likelihood of experiencing cardiovascular events and a 50% elevated incidence of mortality related to cardiovascular disease compared to the general population [6]. This heightened cardiovascular risk in RA is largely attributed to the premature development of atherosclerosis. This process stems from a complex interplay between conventional cardiovascular risk elements and factors intrinsic to RA, such as persistent systemic inflammation and the use of certain medications [7, 8]. The chronic inflammation associated with RA disrupts insulin sensitivity, alters body composition, and adversely affects lipid profiles, contributing to a metabolic syndrome that accelerates atherogenesis [9].

RA and coronary artery disease (CAD) share numerous risk factors, though some present paradoxically in the RA population. While traditional factors like smoking, hypertension, diabetes, and hyperlipidemia are associated contributors [10], smoking is a particularly significant shared risk factor, implicated in both the pathogenesis of RA and the development of CAD [11–15]. Furthermore, RA exhibits a “lipid paradox,” where very low LDL-C levels (below 70 mg/dL) are linked to a remarkably elevated risk of CVD events [16, 17], and an “obesity paradox,” where a higher BMI is associated with reduced cardiovascular mortality compared to normal BMI [18, 19]. Other factors, such as increased insulin resistance [18] and elevated reactive oxygen species (ROS) [20], also contribute to endothelial dysfunction.

The management of RA itself can influence CAD risk. A meta-analysis revealed that individuals with rheumatoid arthritis (RA) who used non-steroidal anti-inflammatory drugs (NSAIDs) and corticosteroids have 18% and 47% increase in cardiovascular event risk [21]. Conversely, Disease-Modifying Anti-rheumatic Drugs (DMARDs), notably methotrexate, have been associated with a decrease of 18% in the risk of CAD [22].

While the association between RA and CAD is well-established globally, this study provides a vital contribution to the global literature from the Eastern Mediterranean, a region consistently underrepresented in large-scale RA cohort studies. Furthermore, the Palestinian cohort serves as a valuable case study for understanding RA-associated cardiovascular risk in many low- and middle-income countries (LMICs). These settings often face a “double burden” of disease: a high prevalence of traditional cardiovascular risk factors coexisting with high, often sub-optimally controlled, RA disease activity and limited access to advanced therapies like biologics, a combination characteristic of many resource-constrained healthcare systems worldwide.

## Methods

### Study design and setting

This cross-sectional study was conducted between March 2024 and September 2024. Participants were recruited from multiple rheumatology clinics and hospitals located in the North, Middle, and South of the West Bank, Palestine, to ensure a representative sample. The study aimed to assess the prevalence of coronary artery disease (CAD) in patients with rheumatoid arthritis (RA) and to identify associated risk factors.

### Study population and sample size

The study population consisted of patients with a confirmed diagnosis of RA. Based on an estimated 10,000 RA patients in the region, a sample size of 370 was calculated to achieve a 95% confidence level with a 5% margin of error, using a standard formula with finite population correction.

384 Patients with Rheumatoid Arthritis were consecutively recruited from four major specialized rheumatology clinics located across the West Bank, Palestine (Bethlehem, Nablus, Hebron, Ramallah) and from Al-ahli hospital.

### Inclusion and exclusion criteria

Inclusion criteria for this study were: [23] patients aged 18 years or older; [24] a confirmed diagnosis of RA according to the 2010 American College of Rheumatology (ACR)/European League Against Rheumatism (EULAR) classification criteria. Exclusion criteria were: [23] a primary diagnosis of another inflammatory joint disease or rheumatological autoimmune disease (e.g., systemic lupus erythematosus, psoriatic arthritis) [24] any severe acute or chronic medical or psychiatric condition that, in the judgment of the investigator, would make the patient inappropriate for study participation.

### Data collection and variables

Data were collected through a structured questionnaire, patient interviews, and a review of medical records. A comprehensive set of variables was collected, including:

- **Demographic and clinical data**: Age, gender, blood pressure, smoking status, body mass index (BMI), and physical activity level.
- **Comorbidities and family history**: Presence of diabetes mellitus, dyslipidemia, and a family history of cardiovascular diseases.
- **RA-specific data**: Duration of disease, medication history (type of medication: DMARDs, biologics, NSAIDs, steroids), and disease activity. Anti-CCP antibody status was determined using standard second-generation ELISA protocols performed at the certified central laboratories of the participating hospitals. Titers were interpreted according to the specific reference ranges validated by each laboratory. In accordance with the manufacturer’s reference values, titers < 20 U/mL were considered negative, titers from 20 U/mL to 60 U/mL were classified as ‘low-positive,’ and titers > 60 U/mL were classified as ‘high-positive.
- **Laboratory and imaging data**: Laboratory results included erythrocyte sedimentation rate (ESR), C-reactive protein (CRP), rheumatoid factor (RF), and anti-cyclic citrullinated peptide (anti-CCP) antibodies.
- **Disease activity assessment**: Disease activity was quantified using the Disease Activity Score 28 (DAS28). The DAS28 is a composite index used to measure RA disease activity, combining a 28-tender and swollen joint count, an inflammatory marker (ESR or CRP), and a patient’s global health assessment on a visual analog scale (VAS). The resulting score is used to classify disease activity into one of four categories: remission (DAS28 < 2.6), low disease activity (2.6 ≤ DAS28 ≤ 3.2), moderate disease activity (3.2 < DAS28 ≤ 5.1), and high disease activity (DAS28 > 5.1).
- **Smoking**: “Smoking status was classified as current smoker (yes) vs. non- smoker/ex-smoker (no).
- **Physical activity**: “Physical activity was self-reported and categorized as low, moderate, or high based on the patient’s daily activity level.”

### Primary outcome definition

The primary outcome, prevalent coronary artery disease (CAD), was defined as a composite endpoint. A patient was considered positive for CAD if a review of their official medical records confirmed a documented history of one or more of the following: [1] acute myocardial infarction, as defined by the treating physician; [24] a coronary revascularization procedure, including either percutaneous coronary intervention (PCI) or coronary artery bypass grafting (CABG); or angiographic evidence of obstructive coronary artery disease was defined as ≥ 50% luminal stenosis in at least one major epicardial coronary artery, according to ACC/AHA guideline criteria [25].

### Statistical analysis

All data were entered and analyzed using SPSS Statistics for Windows, Version 28.0. Descriptive statistics (frequencies, percentages, means, median, and IQR) were used to summarize the characteristics of the participants. Bivariate analyses, including the Chi-squared test for categorical variables and for continuous variables independent t-tests were used for normal distributions data and Mann-Whitney U tests for non-normal distributions data to assess associations between potential risk factors and the presence of CAD. Finally, we fitted a Poisson regression with robust standard errors to identify independent correlates of CAD prevalence. All variables listed under ‘Data Collection and Variables’ were initially considered for inclusion in the multivariable model, including those that were ultimately not statistically significant in the final adjusted model, such as hypertension, diabetes mellitus, smoking status, and DMARD use. We report prevalence ratios (PRs) with 95% confidence intervals and two-sided p values, using α = 0.05.

## Results

**Fig. 1:**
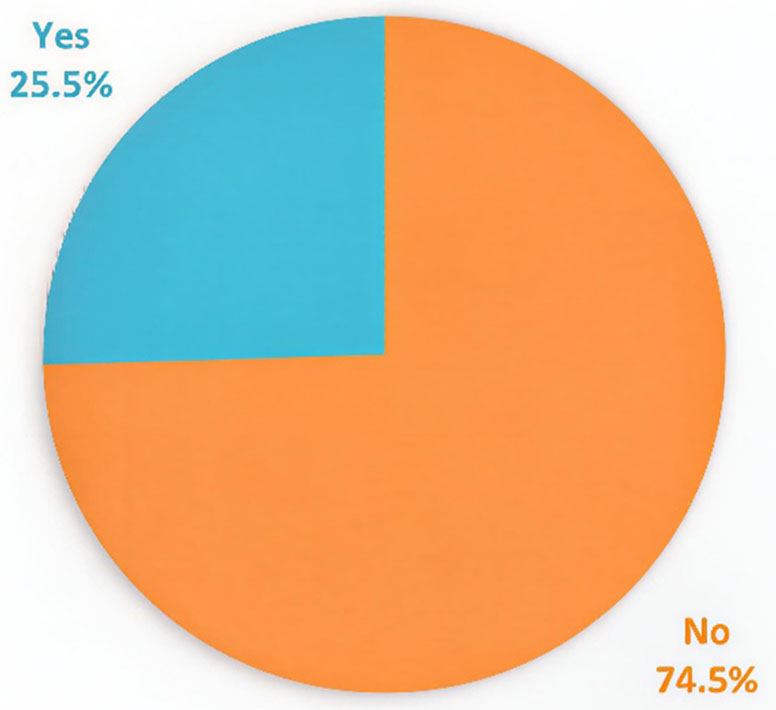
Prevalence of CAD among RA patients

A total of 384 patients with a confirmed diagnosis of rheumatoid arthritis were included in the analysis. The overall prevalence of coronary artery disease (CAD) in this cohort was 25.5%**(**Fig. 1**).**

As shown in Table 1, the cohort presented a substantial burden of traditional cardiovascular risk factors. The prevalence of hypertension was 36.5% and diabetes mellitus was 34.1%. The mean Body Mass Index (BMI) was 31.3 kg/m², indicating a high rate of obesity.

**Table 1:** Sociodemographic characteristics among RA patients

Variable	mean ± SD		median (25%–75%)	
Age	51.1 ± 14.9		51 (42–61)	
BMI (kg/m^2^)	31.3 ± 32.3		28.5 (25.85–31.3)	
Duration of RA	8.0 ± 7.0		5 (2–13)	
DAS28 3 SCORE	5.03 ± 1.2		5.24 (4.355–5.82)	

Variable	**Category**	**Frequency**		**Percent**
Gender	Female	289		75.3
	Male	95		24.7
Physical Activities	Low	160		41.7
	Moderate	162		42.2
	High	62		16.1
Hypertension	No	244		63.5
	Yes	140		36.5
Diabetes	No	253		65.9
	Yes	131		34.1
Dyslipidemia	No	291		75.8
	Yes	93		24.2
Smoking	No	313		81.5
	Yes	71		18.5
Family History CAD	No	282		73.4
	Yes	102		26.6

RA-specific disease characteristics are detailed in Table 2. The patient population exhibited high disease activity, with a mean Disease Activity Score 28 (DAS28) of 5.03. The use of biologic therapies was limited, with only 7% of the cohort receiving them.

**Table 2:** Laboratory and treatment characteristics of RA patients

Variable	Category	Frequency	Percent
CRP	Below 5	157	40.9
	5–10	63	16.4
	More than 10	164	42.7
RF Titer	Negative	201	52.3
	Low-positive	29	7.6
	High-positive	154	40.1
Anti-CCP	Negative	219	57
	Low-positive	112	29.2
	High-positive	53	13.8
Biological	NO	357	93
	YES	27	7
DMARD	NO	99	25.7
	YES	286	74.3
NSAID	NO	230	59.9
	YES	154	40.1
Steroid	NO	178	46.4
	YES	206	53.6

The bivariate analysis of factors associated with CAD is presented in Table 3. A significantly lower prevalence of CAD was observed among patients receiving treatment with disease-modifying antirheumatic drugs (DMARDs) compared to those not on DMARDs (21.4% vs. 37.4%). A non-linear relationship was also noted with anti-cyclic citrullinated peptide (anti-CCP) antibody status; the low-positive anti-CCP group had the highest prevalence of CAD at 42.9%, compared to 15.1% in the high-positive group.

**Table 3:** Distribution of CAD across sociodemografic, lab & treatment factor among RA patients

Variable		CAD		*P*-VALUE
		No	Yes	
Age	median (25%–75%)	49 (40-59.3)	59 (46.5–65.3)	< 0.001
BMI (kg/m^2^)		28 (25–31)	29.4 (27.3–32.9)	< 0.001
Duration of RA		5 (2–10)	9.5 (3-2.25)	< 0.001
DAS28 3 SCORE		4.9 (4.1–5.7)	5.7 (5.1–6.2)	< 0.001
		**N (%)**		
Gender	Female	207 (71.6)	82 (28.4)	0.025
	Male	79 (83.2)	16 (16.8)	
Physical Activities	Low	115 (71.9)	45 (28.1)	0.404
	Moderate	121 (74.7)	41 (25.3)	
	High	50 (80.6)	12 (19.4)	
Hypertension	No	203 (83.2)	41 (16.8)	< 0.001
	Yes	83 (59.3)	57 (40.7)	
Diabetes	No	200 (79.1)	53 (20.9)	0.004
	Yes	86 (65.6)	45 (34.4)	
Dyslipidemia	No	232 (79.7)	59 (20.3)	< 0.001
	Yes	54 (58.1)	39 (41.9)	
Smoking	No	227 (72.5)	86 (27.5)	0.065
	Yes	59 (83.1)	12 (16.9)	
Family History CAD	No	234 (83)	48 (17)	< 0.001
	Yes	52 (51)	50 (49)	
CRP	Below 5	134 (85.4)	23 (14.6)	< 0.001
	5–10	49 (77.8)	14 (22.2)	
	More than 10	103 (62.8)	61 (37.2)	
RF Titer	Negative	153 (76.1)	48 (23.9)	< 0.001
	Low-positive	29 (100)	0 (0)	
	High-positive	104 (67.5)	50 (32.5)	
Anti-CCP	Negative	177 (80.8)	42 (19.2)	< 0.001
	Low-positive	64 (57.1)	48 (42.9)	
	High-positive	45 (84.9)	8 (15.1)	
Biological	NO	269 (75.4)	88 (24.6)	0.155
	YES	17 (63)	10 (37)	
DMARD	NO	62 (62.6)	37 (37.4)	0.002
	YES	224 (78.6)	61 (21.4)	
NSAID	NO	170 (73.9)	60 (26.1)	0.756
	YES	116 (75.3)	38 (24.7)	
Steroid	NO	127 (71.3)	51 (28.7)	0.191
	YES	159 (77.2)	47 (22.8)	

It is noteworthy that the small number of patients (*N* = 29) classified as having a low-positive RF titer were found to have a 0% prevalence of CAD. This finding should be interpreted cautiously, potentially reflecting a statistical anomaly due to the smaller sample size within this specific subgroup.

Table 4 presents the multivariable Poisson regression model identifying independent correlates of coronary artery disease. After adjusting for potential confounders, several factors remained significantly associated with CAD. Traditional risk factors, including increasing age, a diagnosis of dyslipidemia, and a first-degree family history of CAD, were all strongly linked to a higher prevalence of CAD.

**Table 4:** Multivariable Poisson regression model with robust estimators for factors affecting CAD among RA patients

Variable	PR	95% CI	*p*-value
Age	1.017	1.004–1.031	0.01
BMI (kg/m^2^)	0.992	0.987–0.998	0.011
DAS28 3 SCORE	1.309	1.118–1.531	< 0.001
Gender (Ref: Female)	0.807	0.512–1.27	0.353
Hypertension (Ref: No)	1.246	0.868–1.788	0.233
Dyslipidemia (Ref: No)	1.412	1.023–1.95	0.036
Family History CAD (Ref: No)	2.231	1.602–3.108	< 0.001
CRP (Ref: Below 5)			
5–10	1.927	1.093–3.397	0.023
More than 10	2.108	1.378–3.227	< 0.001
Anti-CCP (Ref: High-positive)			
Low-positive	1.811	1.074–3.054	0.026
Negative	1.346	0.801–2.262	0.261
Biological (Ref: No)	1.33	0.791–2.238	0.282
DMARD (Ref: No)	0.756	0.541–1.056	0.101

Crucially, as detailed in Table 4, markers of systemic inflammation were also potent independent correlates. Higher RA disease activity, as measured by the DAS28 score, was independently associated with an increased prevalence of CAD (Prevalence Ratio = 1.309). Similarly, elevated levels of C-reactive protein (CRP) were strongly associated with CAD in a dose-dependent manner, with a PR of up to 2.108 for patients with CRP levels greater than 10 mg/L.

The paradoxical findings from the bivariate analysis were maintained in the multivariable model. Low-positive anti-CCP status remained a significant independent factor associated with CAD (PR = 1.811). Additionally, a modest but statistically significant inverse association was found between BMI and CAD prevalence (PR = 0.992), consistent with the ‘obesity paradox’ described in this population.

## Discussion

This study aimed to determine the prevalence of coronary artery disease (CAD) among patients with rheumatoid arthritis (RA) in Palestine and to identify the associated risk factors. The principal finding is a clinically significant CAD prevalence of 25.5% within this cohort. This high burden of cardiovascular comorbidity is driven by a complex interplay of both traditional cardiovascular risk factors and factors intrinsic to RA.The multivariable analysis revealed that increasing age, dyslipidemia, and a first-degree family history of CAD were independently associated with the presence of CAD. .Crucially, markers of systemic inflammation, specifically higher disease activity as measured by the Disease Activity Score 28 (DAS28) and elevated levels of C-reactive protein (CRP), also emerged as potent and independent correlates of CAD.

These findings provide robust, real-world evidence from a previously unstudied population, suggesting that RA may actively contribute to the atherogenic process. The independent association of DAS28 (Prevalence Ratio = 1.309) and CRP (PR up to 2.108 for levels > 10 mg/L) substantiates the “inflammation hypothesis” of atherosclerosis within the Palestinian context.This dual-pathway model, where both traditional metabolic insults and persistent systemic inflammation independently may drive vascular pathology, underscores the heightened vulnerability of this patient population. The data suggest that the chronic inflammatory milieu characteristic of RA likely lowers the threshold at which traditional risk factors induce vascular damage. Systemic inflammation is known to promote endothelial dysfunction, a critical initiating step in atherosclerosis [26]. A dysfunctional endothelium is more susceptible to the deposition of lipoproteins, even at concentrations that might be considered borderline in a non-inflammatory state. Therefore, the 1.4-fold increased risk associated with dyslipidemia in this cohort may be amplified by the high background inflammation, as evidenced by a mean DAS28 of 5.03. This implies that conventional thresholds for managing traditional risk factors may be insufficient for patients with active RA.

Furthermore, The 25.5% prevalence of CAD observed in our Palestinian cohort is alarmingly high when contextualized with data from the broader Middle East. Studies from Saudi Arabia, for example, have reported a substantially lower prevalence of ischemic heart disease (7.4%) and overall cardiovascular disease (CVD) (6.7%) in their RA cohorts [27]. Similarly, a study from the United Arab Emirates found that while RA patients had a 3.9-fold increased odds of ischemic heart disease compared to controls, the reported baseline prevalence in other regional cohorts appears lower than our finding [28]. This discrepancy suggests that the cardiovascular burden in RA is not uniform across the region and highlights the critical importance of local, population- specific data for informing public health strategy. Several factors may contribute to the higher prevalence observed in Palestine. First, our cohort demonstrated high disease activity, with a mean DAS28 of 5.03, which is a potent factor associated with of cardiovascular risk. Second, the burden of traditional risk factors was substantial, with high rates of hypertension (36.5%), diabetes mellitus (34.1%), and obesity (mean BMI of 31.3 kg/m²), which may exceed those of comparative regional cohorts [27]. Finally, differences in healthcare systems, access to advanced therapies, and socioeconomic determinants of health may also play a significant role.

On a global scale, our findings align more closely with reports from other low- and middle-income country (LMIC) settings. For instance, a systematic review of Latin American studies reported a CVD prevalence of 35.3% in RA patients, suggesting that the burden observed in Palestine is within the range seen in other resource-constrained regions [29]. This study contributes a vital data point from the Eastern Mediterranean, an area underrepresented in the global RA literature. The combination of high CAD prevalence and limited use of biologic therapies (only 7% of our cohort received them) may represent a common challenge in LMICs facing a “double burden” of communicable and non-communicable diseases. This frames the study’s relevance beyond its local context, offering a window into the challenges of managing RA-associated cardiovascular risk in settings where access to optimal anti-inflammatory treatment may be limited.

In this context, Inflammation as the Central Engine of Atherogenesis The strong, dose-dependent association between CRP levels and CAD prevalence, alongside the independent predictive power of the DAS28 score, suggest that systemic inflammation at the center of the heightened cardiovascular risk in this cohort. This aligns with extensive mechanistic evidence establishing RA as a state of accelerated atherosclerosis [26]. Chronic inflammation, driven by pro-inflammatory cytokines such as tumor necrosis factor-α (TNF-α) and interleukin-6 (IL-6), promotes every stage of the atherosclerotic process, from endothelial dysfunction and leukocyte adhesion to lipid oxidation, foam cell formation, and the eventual instability and rupture of atherosclerotic plaques [30]. The clinical relevance of controlling this inflammatory driver is underscored by studies demonstrating that achieving low disease activity (DAS28 ≤ 3.2) significantly reduces the risk of cardiovascular events in RA patients [31]. 

In association, One of the most intriguing and unexpected findings of this study is the non-linear relationship observed between anti-cyclic citrullinated peptide (anti-CCP) antibody status and CAD. The results indicate that the low-positive anti-CCP group had the highest prevalence of CAD (42.9%), and this status remained an independent factor in the multivariate model (PR = 1.811). In contrast, the high-positive group, typically associated with more severe articular disease, exhibited a much lower CAD prevalence of 15.1%.This counterintuitive result challenges the simplistic assumption that higher autoantibody titers uniformly equate to greater systemic risk and suggests a more complex immunopathological relationship.

This hypotheses may explain this observation. It is confounding by indication.High-titer anti-CCP is a well-established marker for aggressive, erosive RA, which often prompts clinicians to initiate earlier and more aggressive anti-inflammatory treatment [32]. This intensive therapy, by effectively suppressing systemic inflammation, may confer a cardiovascular benefit that masks the inherent risk associated with a high-titer serological profile. This hypothesis is strongly supported by our finding that patients receiving disease-modifying antirheumatic drugs (DMARDs) had a significantly lower prevalence of CAD compared to those not on DMARDs (21.4% vs. 37.4%). Conversely, patients with low-titer anti-CCP may be perceived as having a “milder” disease phenotype, potentially leading to less aggressive management and allowing persistent, low-grade inflammation to potentially contribute to athero genesis over time.

In addition, The multivariate model revealed a statistically significant, albeit modest, inverse association between Body Mass Index (BMI) and CAD prevalence (PR = 0.992), an observation often termed the “obesity paradox”. It is crucial to interpret this finding with extreme caution. This statistical association should not be misconstrued as evidence of a biologically protective effect of obesity in RA. Instead, it is likely reflects reverse causation and confounding by disease severity. In chronic inflammatory conditions like RA, unintentional weight loss, or rheumatoid cachexia, is a well-recognized marker of severe, uncontrolled inflammation, poor functional status, and a high catabolic state [33]. Therefore, in a cross-sectional analysis, a lower BMI may paradoxically identify the sickest individuals who have experienced significant disease related weight loss and are consequently at the highest risk for adverse outcomes, including cardiovascular events and mortality.

This interpretation is strongly supported by longitudinal studies in RA cohorts, which have demonstrated that it is weight loss, rather than a low BMI per se, that is the strong predictor of death [34]. Indeed, patients with a low current BMI who have a history of obesity are at the greatest mortality risk, highlighting the danger of illness-induced weight loss [34]. Although our study lacks longitudinal weight data, this external evidence provides the most plausible explanation for our findings. This correct interpretation is vital to prevent the dangerous clinical conclusion that weight gain could be beneficial for RA patients.

Overall, The findings of this study have profound and immediate implications for clinical practice and health policy in Palestine. The high prevalence of CAD necessitates a paradigm shift towards proactive and integrated cardiovascular prevention as a core component of RA management.

For clinicians, this requires moving beyond a sole focus on articular manifestations. A “dual- pronged” therapeutic strategy is warranted, simultaneously targeting RA disease activity and aggressively managing traditional cardiovascular risk factors. Systematic screening for hypertension, dyslipidemia, and diabetes should be standard practice from the time of RA diagnosis and at regular intervals thereafter. Given the inflammatory amplification of risk, clinicians should consider adopting lower thresholds for initiating lipid-lowering or antihypertensive therapies than in the general population. Furthermore, the use of a modified cardiovascular risk calculator, such as applying the 1.5 multiplication factor to standard scores as recommended by the European League Against Rheumatism (EULAR) for RA patients with risk enhancers, should be strongly considered to more accurately stratify risk [35]. 

For health systems and policymakers, this study exposes a critical gap in care coordination. The management of patients with RA and high cardiovascular risk cannot occur in silos. The data compellingly argue for the development of integrated care models that bridge rheumatology and cardiology. This could take the form of joint clinics, shared care pathways, or enhanced cross- disciplinary training and communication protocols. National clinical guidelines specific to cardiovascular risk management in Palestinian RA patients should be developed to standardize care and improve outcomes.

However, This study has several notable strengths. It is the first to systematically quantify the prevalence and risk factors for CAD in a Palestinian RA cohort, filling a significant knowledge gap in the Eastern Mediterranean region. The multicenter design, which recruited patients from various clinics across the West Bank, coupled with a robust sample size (*N* = 370), enhances the potential generalizability of the findings to the broader Palestinian RA population. Furthermore, the use of standardized and validated measures for RA disease activity, such as the DAS28,ensures methodological rigor and facilitates comparison with international literature.

Nevertheless, Our study has several methodological limitations. First, due to the cross-sectional design, we are unable to establish causality between observed risk factors (e.g., CRP levels, Anti-CCP status) and the development of Coronary Artery Disease (CAD); thus, only associations can be reported. Second, the reliance on medical records and patient self-report for CAD diagnosis introduces a potential for recall bias or misclassification. Third, the patient cohort was recruited from specialized clinics, which may result in selection bias towards more severe or chronic disease, potentially overestimating the true CAD prevalence in the wider Palestinian RA population. Finally, while our regression model adjusted for major confounders, the possibility of residual confounding from unmeasured factors (e.g., cumulative inflammatory burden, specific genetic markers) remains.

The findings from this study lay the groundwork for a critical research agenda. The most pressing need is for the establishment of a prospective, longitudinal cohort of Palestinian RA patients. Such a study would be invaluable for elucidating causal relationships, tracking the evolution of risk factors over time, and identifying predictors of incident cardiovascular events.

Building on our findings, future research should also focus on designing and testing targeted interventions. Randomized controlled trials of integrated care models, such as nurse-led clinics for comprehensive cardiovascular risk management in RA patients, are needed to determine their effectiveness in reducing cardiovascular morbidity and mortality in this setting. Finally, further mechanistic studies are warranted to explore the paradoxical associations observed with anti-CCP antibodies and BMI, which may involve detailed immunological profiling and body composition analyses to investigate the role of sarcopenia.

## Conclusion

This study reveals a high prevalence of coronary artery disease among Palestinian patients with rheumatoid arthritis, a burden driven by a potent combination of traditional metabolic risk factors and RA-related systemic inflammation. The findings underscore the urgent need to integrate proactive cardiovascular disease prevention into the standard of care for RA in Palestine and similar settings worldwide. Rheumatoid arthritis should be recognized as a cardiovascular risk-equivalent condition, warranting aggressive, holistic management to mitigate its substantial impact on morbidity and mortality. Future research should focus on establishing a prospective, longitudinal cohort to elucidate causal relationships and on designing and testing targeted interventions, such as integrated care models for comprehensive cardiovascular risk management.

## Supplementary Information

Below is the link to the electronic supplementary material.

Supplementary Material 1

## Data Availability

The datasets generated and/or analyzed during the current study are not publicly available due to patient privacy restrictions but are available from the corresponding author on reasonable request.

## Abbreviations

ACR: American College of Rheumatology
Anti-CCP: Anti-cyclic citrullinated peptide
BMI: Body Mass Index
CAD: Coronary Artery Disease
CRP: C-reactive protein
CVD: Cardiovascular Disease
DAS28: Disease Activity Score 28
DMARDs: Disease-Modifying Antirheumatic Drugs
EULAR: European League Against Rheumatism
NSAIDs: Non-steroidal anti-inflammatory drugs
RA: Rheumatoid Arthritis
RF: Rheumatoid Factor
