# Supplementary material for: The prevalence of coronary artery disease in rheumatoid arthritis patients in Palestine: a cross-sectional study
Source: BMC Rheumatol. 2026 Jan 20;10:15. doi: 10.1186/s41927-026-00613-3 (PMC12906049; doi:10.1186/s41927-026-00613-3)
Supplement: Supplementary file 1 — Supplementary Material 1 [file 41927_2026_613_MOESM1_ESM.docx]

| **Variable** | **Tolerance** | **VIF** |
| --- | --- | --- |
| Age | 0.824 | 1.214 |
| BMI | 0.979 | 1.021 |
| DAS28 3 SCORE | 0.870 | 1.150 |
| Gender | 0.964 | 1.037 |
| Hypertension | 0.739 | 1.354 |
| Dyslipidemia | 0.858 | 1.165 |
| Family History CAD | 0.891 | 1.122 |
| CRP | 0.900 | 1.111 |
| Anti-CCP | 0.943 | 1.061 |
| Biological | 0.923 | 1.083 |
| DMARD | 0.954 | 1.048 |

Table S1. Collinearity statistics (tolerance and variance inflation factor) for independent variables in the multivariable Poisson regression model
